# Evaluation of Anticonvulsant Effect of Aqueous and Methanolic Extracts of Seven *Inula* Species 

**DOI:** 10.22037/ijpr.2019.15509.13151

**Published:** 2019

**Authors:** Maryam Ranjbar Ekbatan, Mona Khoramjouy, Babak Gholamine, Mehrdad Faizi, Shamim Sahranavard

**Affiliations:** a *Islamic Azad University, Pharmaceutical Sciences Branch, Tehran, Iran. *; b *Department of Pharmacology and Toxicology, School of Pharmacy, Shahid Beheshti University of Medical Sciences, Tehran, Iran. *; c *Department of Pharmacology, School of Medicine, Shahid Beheshti University of Medical Sciences, Tehran, Iran, *; d *Department of Traditional Pharmacy, School of Traditional Medicine, Shahid Beheshti University of Medical Sciences, Tehran, Iran.*

**Keywords:** Anticonvulsant, Pentylentetrazole, Maximal electroshock, Inula, Hypnotic.

## Abstract

In Iranian traditional medicine, *Inula* species have been used for the treatment of seizure. In this study we decided to investigate the anticonvulsant activity of seven species from this genus to find an effective remedy for seizure with less adverse effects compared to the available medicines. Aqueous and methanolic extracts of *Inula britannica*, *Inula helenium*, *Inula viscidula*, *Inula oculus-christi*, *Inula aucheriana*, *Inula thapsoides*, and *Inula salicina* were prepared and their antiepileptic activity was investigated by maximal electroshock (MES) and pentylentetrazole (PTZ) tests on Male NMRI Albino mice. Diazepam was used as positive control in both tests. In addition, two extracts with the best anticonvulsant activities were selected and their sedative and hypnotic effects were evaluated using open field and righting reflex tests, respectively. The effects of the both extracts on memory and motor coordination were also assessed by step-through passive avoidance and rotarod tests, respectively. Aqueous extract of *Inula britannica* and *Inula viscidula* showed the best activity in MES model and their ED_50_ (with 95% confidence interval) was 19.5 (7.9~48.5) mg/kg and 12.7(10.0~16.3) mg/kg, respectively. None of the extracts showed noticeable anticonvulsant effects in the PTZ model. The active extracts also showed sedative-hypnotic effects in righting reflex and open field tests. Furthermore, both extracts did not affect the memory and motor coordination in the experimental models. The anticonvulsant and sedative activities of the extracts were antagonized by flumazenil, indicating that benzodiazepine receptors are probably involved in the effects. This study indicates that *Inula britannica* and *Inula viscidula* are good candidates for further phytochemical and mechanistic studies in order to find anticonvulsant and sedative-hypnotic compounds with less adverse effect on memory and motor coordination.

## Introduction

Epilepsy is a common chronic neurological disorder that affects approximately 1-2% of the global population (1). Despite many existing anticonvulsant agents, about one-third of the epileptic patients are still suffering from seizures refractory to the standard therapies (2). Moreover, most antiepileptic drugs may cause many unwanted effects including tremor, sedation, memory problems, and other cognitive and motor impairments (3). Discovery of many drugs is dependent on natural products derived from folk medicine. One of the alternative sources for discovery of antiepileptic medicines can be natural products with better safety and efficacy profiles and possible novel chemical structures (4,5). As a result, investigation of medicinal plants with anticonvulsant activity is an important goal in the epilepsy research (6).

The genus *Inula* belongs to the Asteracea family with about 100 species in the world (7). The compounds present in the plants of genus *Inula* show wide range of biological and medicinal activities i.e., hepatoprotective, cytotoxic, anticancer, antibacterial, and anti-inflammatory properties (8). These plants are rich sources of a wide variety of bioactive secondary metabolites such as tannis, terpenoids (alantolactone, isoalantolactone, steroids, terpenoids (sesquiterpen lactones, diterpens and triterpenoides) and flavonoids (9). Flavonoids are a family of compounds with affinity to GABA_A_ receptors and have anxiolytic and antiepileptic effects similar to the benzodiazepines (BZDs) (10,11). Moreover, because of their phenolic structure, they can prevent the cellular oxidative process in the central nervous system and can be useful in neurodegenerative disorders (10). Some flavonoids in *Inula britannica* such as patuletin, neptin, axillarin and quercetin modulate GABA_A_ receptors and also act as NMDA receptor antagonists (12,13). Hispidulin, a flavonoid in *Inula oculus-christi* crosses the blood-brain barrier, displays antiepileptic activity and reduces glutamate release. Hispidulin decreases voltage-dependent Ca^+2^ entry, and interferes with the exocytotic machinery release (14). Alantolactone and isoalantolactone are also the major compounds in *Inula* plants especially in *Inula helenium*, with anti-inflammatory, antimicrobial, antifungal, antihelminthic, antiproliferative, and anticonvulsant properties (15, 16, 9, 17) In the present study, seven species of *Inula, *including* I. britannica*, *I. helenium*, *I. viscidula*, *I. oculus-christi, I. aucheriana*, *I. thapsoides* and *I. salicina* were collected, and based on reports in Iranian folk medicine, their antiepileptic effects were evaluated (18).

## Experimental

*Plant material*

The plants were collected from west Azerbaijan, Iran and identified by a qualified botanist at Traditional Medicine and Materia Medica Research Center (TMRC), Shahid Beheshti University of Medical Science, Tehran, Iran.

*Extract Preparation*

The aerial parts of *Inula helenium*, *Inula* *oculus*-*christi*, *Inula* *aucheriana*, and *Inula* *viscidula *and the whole parts of *Inula* *salicina*, *Inula* *thapsoides*, and *Inula* *britannica* were dried in shade and powdered by a mechanical grinder. The powder was stored in an airtight container at 4 ^o^C until the experiment.

*Methanolic extraction*

The Powdered dried materials were macerated in methanol for 24 hours and then filtered through a paper filter. The filtrates were evaporated to dryness and kept at 4 ^o^C until used.

Aqueous extraction:

Powdered dried materials were macerated in water and allowed to shake for 24 hours, then filtered through a paper filter and the filtrates were centrifuged. The maceration process was repeated twice. The filtrates were freeze-dried and kept at 4 ^o^C. 

*Animals*

The male NMRI Albino mice weighting 18-25 grams were used for the tests. The animals were housed in a standard animal house with controlled condition and 12-hour light/dark cycles and all pharmacological experiments were performed between 9:00 to 15:00. Five days prior to the experiments, the animals were handled to habituate with the environment and the experimenter. The animals were selected randomly 30 min before the experiment and allowed to acclimatize with the experiment room before injecting the extracts or vehicle. The extracts (10 mL/kg), flumazenil (Sigma-Aldrich), PTZ (Sigma-Aldrich), diazepam (Sigma-Aldrich), pentobarbital, and midazolam were given *i.p.* as freshly prepared solutions. The aqueous and methanolic extracts were dissolved in water and suspended in water and 1% Twin 80 respectively. We carried out all experiments in according to National Institute of Health (NIH) guide for use and care of laboratory animals and we made an effort to use as less animals as possible in this study.

*Anticonvulsant studies*

Pentylentetrazole (PTZ) and maximal electroshock (MES) models were used to evaluate the anticonvulsant effects of the extracts in groups of 8 mice. PTZ at a dose of 100 mg/kg was used to induce convulsion. This dose leads to death in 100% of the mice (19). Fifteen minutes after the injection of extracts at the doses of 12.5– 800 mg/kg, vehicle (10 mL/kg), or diazepam (1 mg/kg), flumazenil (10mg/kg) as a BZD antagonist were administered, and after 15 min PTZ was injected. After the injection of PTZ (100mg/kg; *i.p.*), we observed the animals for 60 min for occurrence of hind limb tonic extension (HLTE). The dead mice were counted within 24 h (raw data are shown in supplementary data). In the MES model, we injected the extracts at the range of doses 12.5- 800 mg/kg, vehicle (10 mL/kg), or diazepam (1 mg/kg) 30 minu before and flumazenil (10 mg/kg) 15 min before the induction of seizure. The electrodes were connected to the ears of the mice and an alternating electric current (40 Hz, 50 mA) was connected to the animals for 0.2 seconds for induction of hind limb tonic extension HLTE (20, 21). Seizures in mice were manifested as HLTE, and the ability of the extract to prevent this feature was diagnosed as an indicator of the anticonvulsant activity (22, 23). We also observed the animals for mortality for 24 hours following MES.

*Righting reflex test*

The righting reflex test was used for the assessment of the hypnotic effects of the extracts. The test is based on potentiating the pentobarbital induced sleeping time (loss of righting reflex). Thirty minutes after administration of the extracts at the doses of 19.5 and 12.71 mg/kg (ED_50_ calculated from MES test of the aqueous extracts of *I. britannica* and *I. viscidula*.), pentobarbital (40 mg/kg, *i.p.*) was given to induce sleep. The interval between the loss of the reflex and recovery was used as an index for the hypnotic effect. The latency time was recorded as the time interval between pentobarbital injection and start of losing righting reflex. Involvement of BZD receptors in the hypnotic effects of the extracts was evaluated by flumazenil. Flumazenil was given (10 mg/kg, *i.p*.) 15 min after injections of diazepam or the extracts. Subsequently, the righting reflex test was carried out after 30 min (24, 25). 

*Step-through passive avoidance test*

A modification of step-through passive avoidance test was used as an index to measure the effect of the extracts on memory in mice. The apparatus (Malek Teb Co, Tehran) contained a rectangular chamber divided into 2 compartments. One of the compartments was lighted, and the other one was dark. A guillotine door divided the compartments. A grid floor placed through the dark compartment is capable of giving a foot shock. Each animal was located in the lighted compartment and allowed to walk around for 30 min in the training trial. The guillotine door was lifted after 30 seconds, and as the animal entered all four paws in the dark compartment, the door was closed, and the electric shock (0.5 mA, 2 seconds) was given through the grid floor. At the end, the mouse was removed from dark compartment, and returned to the home cage. On the testing date, 24 h after training, the extracts at the doses of 19.5 and 12.71 mg/kg were injected *i.p*. to the mouse 30 min prior to the test. The mouse was returned to the lighted compartment, and the guillotine was lifted after 5 seconds. The time interval between lifting the guillotine and mouse entrance to the dark compartment was recorded. A cut off time of 8 min was applied. Extracts and midazolam (1 mg/kg, as a positive control) were administered 30 min prior to the training trial. Prolongation of latency in entrance to the dark room was used as a parameter of memory recall (26, 27).

*Locomotion activity in mice*

To examine the acute sedative responses to *Inula* extracts, we evaluated the locomotion activity of the animals using open field test 30 min following *i.p.* injection of the extracts at the doses of 19.5 and 12.71 mg/kg. The animals were placed separately in the center of square area of the open field box (40×40×40 cm) and were allowed to discover the unfamiliar surroundings freely. The locomotion activity of the animals were recorded and videotaped for 10 min using a digital camera installed on top of the arena. The distance travelled by each animal, named as total distance moved, was measured as an indication of locomotion activity. Tracking the animals was done by Ethovision XT (Noldus, The Netherlands) software (28, 29). 

*Rotarod test*

We used rotarod test in this study to investigate motor coordination and balance in mice. Briefly, mice were trained to remain on a rolling device with slowly revolving rod with 2 cm diameter at the speed of 10 rpm and acceleration of 7 rpm^2^. Those mice that were able to remain on the rod for 60 seconds or longer were selected and randomly divided into three groups of eight mice. One group received saline, while the other two groups received extracts at the doses of 19.5 and 12.71 mg/kg. Thirty minutes after the injections, animals were placed on the rod. If animals failed to stay on the rod for minimum of 60 seconds, the test was considered positive, meaning there was a deficit of motor coordination (30, 31). 

*Acute toxicity study*

Fiftyfold of the ED_50_ values of the extracts in MES test of the aqueous extract of *I. britannica* and *I. viscidula *(975 and 635 mg/kg respectively) was injected to groups of 8 mice. We observed them for 24 h to see the possible lethal effects and acute toxicity of the extracts (32,33). 

*Statistical analysis*

To determine the ED_50_s of anticonvulsant effects of the extracts, linear regression analysis and SPSS software (Chicago, IL, version 13) were used. Fisher’s exact probability test was used to analyze the difference between the ED_50_s of the extracts in the experimental groups. All data were expressed as mean (with 95% confident limits) and *p*<0.05 was counted as a statistically significant difference. 

Results of locomotion activity, sleep duration, passive avoidance, and rotarod tests were analyzed by one-way ANOVA followed by Tukey’s HSD test using GraphPad Prism software version 5. All values were presented as mean± SEM. Similar to the data analysis of anticonvulsant effect, *p* <0.05 was counted as statistically significant.

## Results

The extraction yields of the two solvents (methanol and water) from 100 g of plant material are presented in Table 1. Maximum extraction yield was 20.54% (grams of extract per 100 g of dried parts) for aqueous extract of *I. aucheriana* and minimum extraction yield was 6.77% for methanolic extract of *I. oculus-christi*.

*Anticonvulsant activity*

The animals (n = 8) were administered with different doses of the aqueous and methanolic extracts (shown in supplementary data). Results showed that based on the mortality of animals in each group, the most protected animals were treated with *Inula britannica* and *Inula viscidula* aqueous extracts with 8 protected animals at the dose of 200 mg/kg in MES test**.**

ED_50_ of the aqueous and methanolic extracts are presented in Table 2. The ED_50_ of diazepam in preventing the mice from HLTE in MES test was 0.98 mg/kg with 95% confidence intervals of 0.69-1.22 mg/kg. Among all the extracts, the aqueous extract of *I. britannica* and *I. viscidula* were the most potent extracts against MES-induced seizure with ED_50_ values of 19.5 and 12.71 mg/kg respectively. Furthermore, the aqueous and methanolic extracts of *I. helenium *and* I. thapsoides*, aqueous extract of *I. salicina* and methanolic extracts of *I. aucheriana* had an ED_50_ values < 70 mg/kg. This indicates that these extracts were slightly less potent than the aqueous extracts of *I. britannica* and *I.* *viscidula*.

As shown in Table 2, most of the extracts did not have potent anticonvulsant effects in the PTZ-induced seizure test. Moreover, the ED_50_ of diazepam in preventing the mice from PTZ-induced seizure was 0.68 mg/kg with the 95% confidence limits of 0.41-0.83 mg/kg.

The comparison of the obtained results from PTZ and MES tests revealed that all of the extracts had lower ED_50_ in MES test than PTZ model of epilepsy. Even the aqueous and methanolic extracts of *I. oculus-christi* and methanolic extract of *I. britannica* and *I. salicina*, which were almost inactive in the PTZ test, showed protective effects against MES induced seizure.

Flumazenil, a well-known benzodiazepine antagonist, prevented the antiepileptic activities of the most potent extracts (aqueous extract of *I. britannica* and *I. viscidula*) in MES test.

*Righting reflex test*

**Figure 1:**
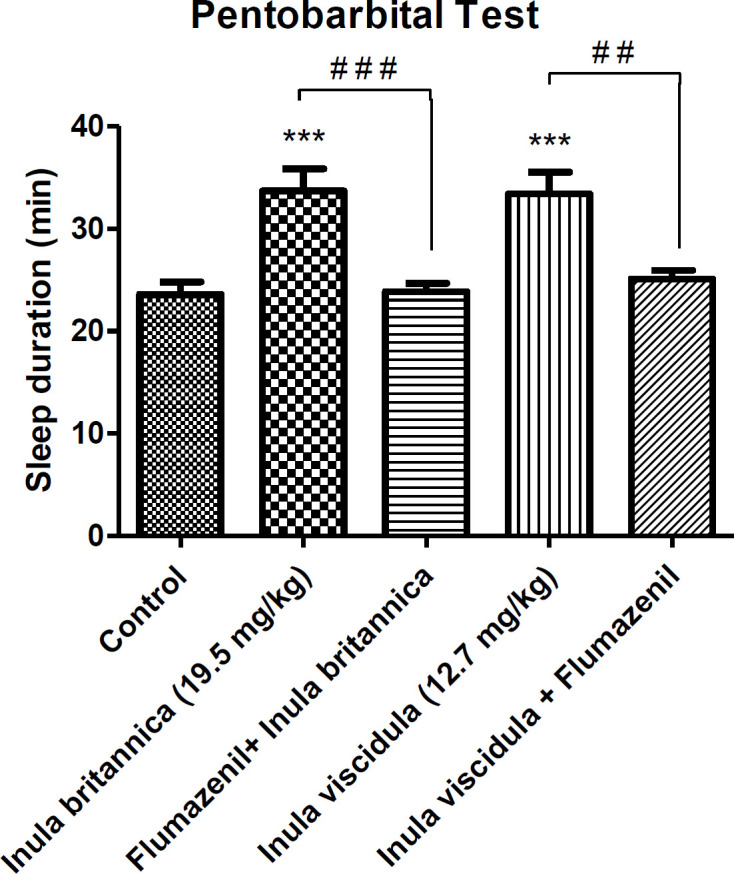
Evaluation of hypnotic effect of the extracts based on the increasing of sleeping time. Data are presented as mean ± SEM. ***p < 0.001 compared to control group; ##*p* < 0.01 and ###*p* < 0.001 compared to the groups shown on the graph

As shown in Figure 1, the aqueous extracts of *I. britannica* and *I. viscidula* significantly prolonged the duration of righting reflex loss. For both extracts, ED_50_ of the extracts in MES test were used. However, the onset of pentobarbital induced sleeping was not affected by the injection of extracts. There was no increase in loss of righting reflex by administration of flumazenil after the injection of the extracts in animals receiving pentobarbital and control group. In other words, flumazenil prevented the hypnotic effects of the extracts.

*Step-through passive avoidance test*

**Figure 2:**
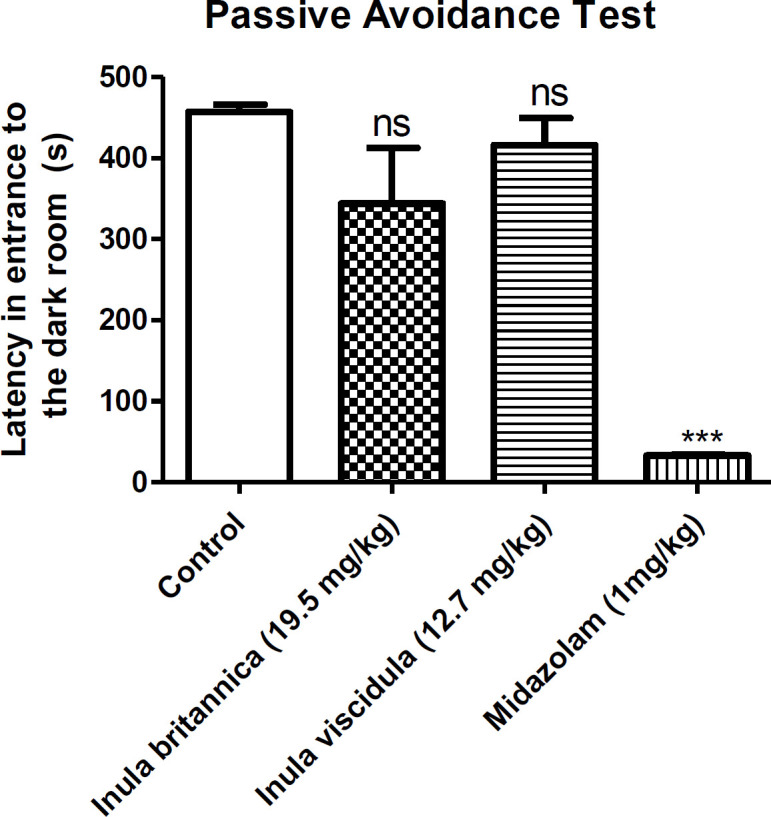
The memory loss effect of the aqueous extracts of *Inula* *britannica* and *Inula* *viscidula* (19.5 and 12.7 mg/kg, i.p.). Data are presented as mean±SEM. ns: not significant, ***p<0.001 compared to the control group

To evaluate the effects of the most potent extracts (in MES test) on memory, the prolongation of latency to entrance to the dark room was used as a parameter of learning and memory. The results of the step-through passive avoidance test are shown in Figure 2.

The results showed that the most potent extracts (at a dose of the ED_50_ in MES test) were not able to significantly change the latency to entrance to the dark room. In other words, the injection of the ED_50_ of extracts 24 hours before experiment did not have negative effects on memory. However, midazolam significantly decreased the latency to entrance to the dark room.

*Locomotor activity*

**Figure 3.**
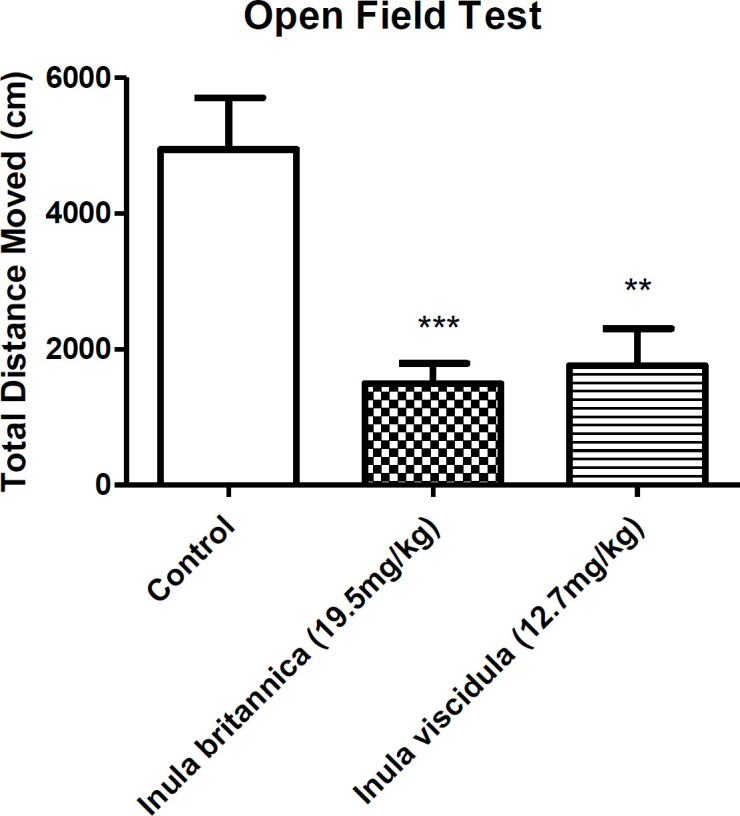
locomotor activity of* Inula britannica* and *Inula viscidula* aqueous extracts (19.5 and 12.7 mg/kg, i.p.) Data are presented as mean±SEM. ***p<0.001, **<0.01 compared to the control group

**Figure 4:**
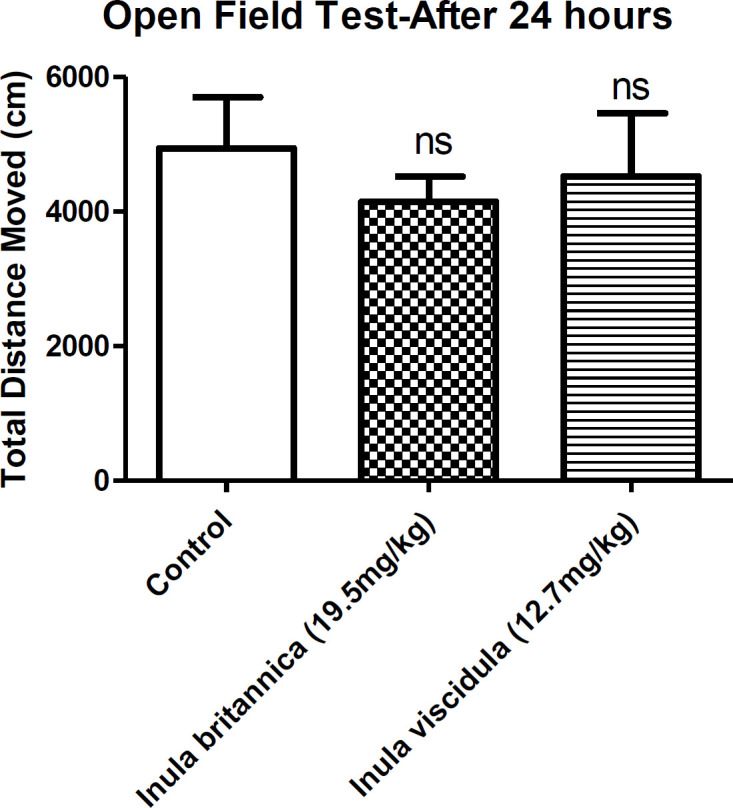
The total distances moved by the animals compared to the control group after 24 h. Data are presented as mean ± SEM. ns: not significant

Locomotor activity was used as an index of sedative properties of the extracts along with loss of righting reflex examination. The results concluded from open field and loss of righting reflex tests showed the sedative-hypnotic activities of the extracts. In the current test the total distances moved by the animals were compared as an indicative of locomotor activity. Locomotor activity of the animals decreased 30 min after injection of the aqueous extracts of *I. britannica* and *I. viscidula* (at dose of the ED_50_ in MES test) (Figure 3). However, after 24 h of the extracts administration, locomotor activity of the animals showed no differences compared to the control group (Figure 4). 

*Neurotoxicity*

**Figure 5:**
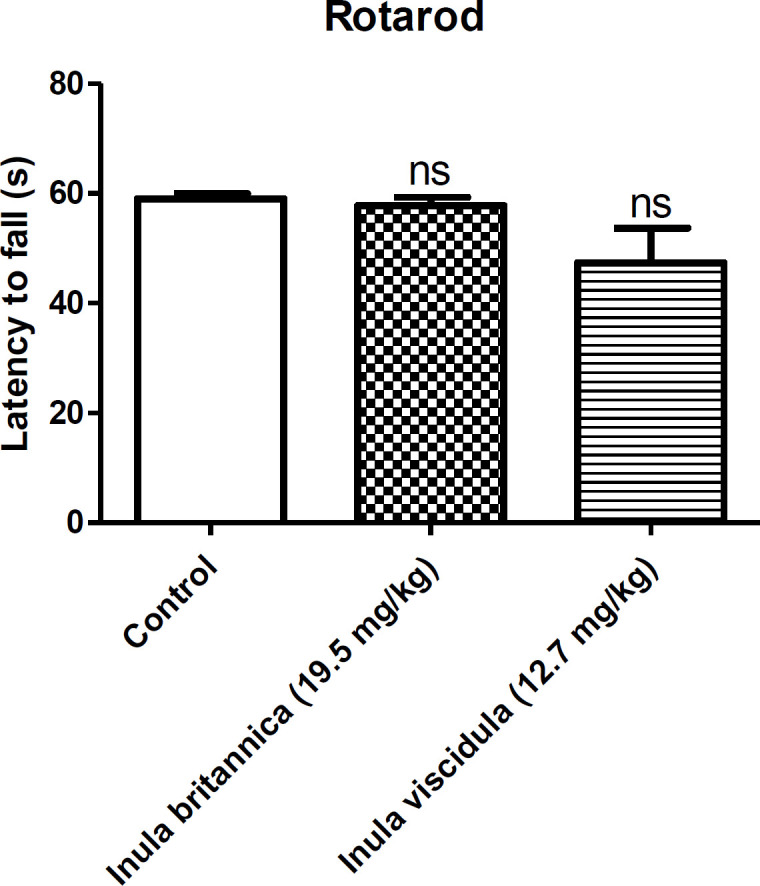
Effects of the aqueous extract of *Inula* britannica and *Inula* viscidula (19.5, 12.7 mg/kg, i.p. respectively) in rotarod test. Data are presented as mean±SEM. ns: not significant

The ED_50_ of the aqueous extracts of *I. britannica* and *I. viscidula* were assessed for their neurotoxicity in mice by rotarod test. Extracts had no effect on motor coordination as shown by the rotarod performance in mice (Figure 5). All mice stayed on the rotarod for 60s without falling during the observation.

*Acute toxicity study*

Lethal and toxic effects were not observed after *i.p.* administration of the aqueous extracts of *I. viscidula* and *I. britannica* at the doses of 635 and 975 mg/kg respectively, suggesting that *I. britannica* and *I. viscidula* were apparently safe when administered intra-peritoneally.

## Discussion

Since a considerable number of people in the world are suffering from seizure, finding new drugs with efficient anticonvulsant activity and with no negative effects on memory and locomotor activity is very important. (34) 

The anticonvulsant effects of *Inula conyza* is indicated in some Iranian traditional medicine references such as *Al-Abniah an Haqaeq al Adwia*, *Canon* (written by Avicenna), *Makhzan ul-Adwia* and *Tuhfat al-Muminin *(18). There are no studies on the anticonvulsant effects of this species and other species in this genus. PTZ and MES tests are standard models used for evaluating anticonvulsant effects of compounds in absence and tonic-clonic generalized seizure, respectively (35). Due to the anticonvulsant effects of *Inula britannica* and *Inula viscidula* in MES test, it can be concluded that these extracts contain compounds with potential values of controlling tonic-clonic generalized seizure. Furthermore, MES test is suitable test for analyzing the anticonvulsant activity of specific drugs, including drugs that enhance gamma-aminobutyric acid (GABA) receptors and drugs that are glutamate receptor antagonists (36). 

The reduction in the effects of extracts as well as diazepam by flumazenil with respect to the susceptibility of MES test in GABA enhancing drugs can lead to the conclusion that their effect is at least in part mediated through benzodiazepine receptors. Effects of the neurotransmitter GABA are enhanced by benzodiazepines at the GABA_A_ receptor, resulting in sedative, hypnotic and anticonvulsant properties as well as motor coordination impairment and memory loss depending on which subtype of GABA_A_ receptor they interact with (37,38). Based on previous studies, drugs that interact with alpha-1 and alpha-5 subunits are more associated with sedation (39) and amnesia respectively (40, 41). In contrast subunits of alpha-2 and alpha-3 tend to be more effective for anxiolytic properties (42). On the other hand, anticonvulsant properties can be made through all subunits (43).

The extracts significantly decreased the total distance moved 30 min after the administration in mice. This indicates the sedative effects of the extracts. Since the extracts increased the duration of pentobarbital-induced sleep in mice, it can be concluded that they may have sedative-hypnotic effects by acting on alpha-1 subunits of GABA_A_ receptors (44). 

The results of the passive avoidance test revealed that the aqueous extracts of *I. britannica* and *I. viscidula* did not have negative effects on memory at doses with anticonvulsant activity. Since memory problem is one of the adverse effects of benzodiazepine agonists which interact with alpha-5 subunit of GABA_A_ receptor (45), this can be an advantage of these extracts compared to benzodiazepines. The active compounds of the extracts may act selectively on the other subunits of GABA_A_ receptors rather than the alpha-5 subunit of the receptors.

We ran the open field test 24 h after the administration of the extracts. The locomotor activity was not reduced compared to the control groups. If the locomotion activity is changed, the entrance time to dark compartment will be changed too. Since extracts did not change the locomotor activity after 24 h, we can suggest that locomotor activity was not effective in entrance time to the dark compartment.

Since the extracts had no effects on motor coordination in the rotarod test, we can conclude that the cause of observed sedative effects may be due to the central mechanism rather than peripheral neuromuscular blockade. In addition, rotarod test is an experiment for screening the neurotoxicity of many compounds (46). The extracts did not show deficit in the rotarod test and they may have no neurotoxicity. The dose of 975 and 635 mg/kg (50 times of ED_50_ values of the extracts in MES test) for *I. britannica* and *I. viscidula* caused neither visible signs of toxicity nor mortality in mice, suggesting their relative safety profile. Obviously, these data indicate an estimate of acute toxicity of extracts and comprehensive toxicological experiments might be necessary for confirming their safety.

Several compounds are collected from different plants with anticonvulsant effects like flavonoids, saponins, and terpenes. Flavonoids show their effects by reducing oxidative stress and affecting GABA_A_ receptors because of their similarity to benzodiazepines (47).

Our experiments show that the ED_50_ of all the aqueous extracts are lower than their methanolic extracts, resulting in their higher potency in tonic-clonic generalized seizure. Therefore, it can be concluded that the effective constituents may be polar rather than semi and nonpolar constituents. Because of the presence of hispidulin in *I. oculus-christi*, and neptin, quercetin, axillarin in *I. Britannica* (48), we can conclude that the anticonvulsant activities, at least to some extent, are related to the flavonoids compounds. Moreover, based on previous studies which is conducted in MES test, the anticonvulsant effects of *I. helenium* may be because of terpenes such as isoalantolactone and alantolactone separated from the plant (49). 

As a result, the aqueous extracts of *I. britannica* and *I. viscidula* have considerable anticonvulsant effects, without any effects on memory. They also have sedative-hypnotic effects that might be useful in some patients with sleep disorders. Absence of negative effects on memory and neurotoxicity are positive characteristics of these extracts in comparison to other anticonvulsant compounds. 

**Table 1:** Extraction yield (gram of extract per 100 g of dried plant material).

**Herbarium number**	**Yield of methanolic extract (%)**	**Yield of aqueous extract (%)**	**Used part**	**Name of the plant**
436-TMRC	8.11	9.64	Aerial part	***Inula helenium***
69- TMRC	7.01	10.35	Whole parts	***Inula salicina***
770- TMRC	7.94	12.56	Whole parts	***Inula thapsoides***
840- TMRC	6.77	11.67	Aerial part	***Inula oculus-christi***
556- TMRC	9.87	20.54	Aerial part	***Inula aucheriana***
554- TMRC	11.13	11.88	Aerial part	***Inula viscidula***
454- TMRC	8.76	12.55	Whole parts	***Inula britannica***

**Table 2:** Anticonvulsant activity of *Inula* extracts in PTZ and MES induced convulsion test

**ED**_50 _ **(mg/kg)** ^ a^		**Extraction solvent**	**Name of the plant**
**PTZ**	**MES**		
>2000	523(335-816)^ b^	Water	***Inula oculus-christi***
>2000	472.3(251.9-885.7)	Methanol	
>2000	41.6 (23-163)	Water	***Inula helenium***
1289	69.15 (115.62-306.2)	Methanol	
>2000	19.5 (7.9-48.5)	Water	***Inula britannica***
>2000	343.9 (287.7-411)	Methanol	
>7000	55.8 (31.5-98.7)	Water	***Inula salicina***
>2000	117.9 (88.47-157)	Methanol	
>2000	12.71 (10-16.3)	Water	***Inula viscidula***
1289	113.8 (85-152.5)	Methanol	
>7000	45.47 (22.3-93)	Water	***Inula thapsoides***
>7000	61.94 (11.59-331.1)	Methanol	
>2000	99.13 (65.6-149.3)	Water	***Inula aucheriana***
>2000	58.05 (37.43-90)	Methanol	
0.68 (0.41-1.22)	0.98 (0.62-1.22)	-	**Diazepam**

^a^ ED_50_ indicates the median effective dose protecting 50% of animals from convulsion

^b^ 95% confidence intervals given in parentheses

**Table 3:** Doses of aqueous and methanolic extracts of *Inula oculus-christi* and number of protected animals from seizure in MES test

**Plant's name**	**Number of animals (each dose)**	**Doses (mg/kg) of extract** **s**	**Number of protected animals (aqueous extract)**	**Number of protected animals (methanolic extract)**
*Inula oculus-christi*	8	50	1	0
		100	2	2
		200	2	3
		400	4	4
		800	4	5

**Table 4.** Doses of aqueous and methanolic extracts of *Inula helenium* and number of protected animals from seizure in MES test

**Plant's name**	**Number of animals (each dose)**	**Doses (mg/kg) of extract** **s**	**Number of protected animals (aqueous extract)**	**Number of protected animals (methanolic extract)**
*Inula helenium*	8	25	0	4
		50	4	4
		100	6	5
		200	7	6
		400	8	7

**Table 5:** Doses of aqueous and methanolic extracts of *Inula Britannica* and number of protected animals from seizure in MES test

**Plant's name**	**Number of animals (each dose)**	**Doses (mg/kg) of extract** **s**	**Number of protected animals (aqueous extract)**	**Number of protected animals (methanolic extract)**
*Inula britannica*	8	12.5	3	4
		25	5	4
		50	6	5
		100	8	6
		200	8	7

**Table 6:** Doses of aqueous and methanolic extracts of *Inula salicina* and number of protected animals from seizure in MES test

**Plant's name**	**Number of animals (each dose)**	**Doses (mg/kg) of extract** **s**	**Number of protected animals (aqueous extract)**	**Number of protected animals (methanolic extract)**
*Inula salicina*	8	25	3	2
		50	4	2
		100	6	4
		200	6	5
		400	8	6

**Table 7:** Doses of aqueous and methanolic extracts of *Inula viscidula* and number of protected animals from seizure in MES test

**Plant's name**	**Number of animals (each dose)**	**Doses (mg/kg) of extract** **s**	**Number of protected animals (aqueous extract)**	**Number of protected animals (methanolic extract)**
*Inula viscidula*	8	12.5	4	0
		25	5	3
		50	6	4
		100	7	5
		200	8	6

**Table 8:** Doses of aqueous and methanolic extracts of *Inula thapsoides* and number of protected animals from seizure in MES test

**Plant's name**	**Number of animals (each dose)**	**Doses (mg/kg) of extract** **s**	**Number of protected animals (aqueous extract)**	**Number of protected animals (methanolic extract)**
*Inula thapsoides*	8	25	2	0
		50	5	3
		100	6	6
		200	7	7

**Table 9:** Doses of aqueous and methanolic extracts of* Inula aucheriana* and number of protected animals from seizure in MES test

**Plant's name**	**Number of animals (each dose)**	**Doses (mg/kg) of extract** **s**	**Number of protected animals (aqueous extract)**	**Number of protected animals (methanolic extract)**
*Inula aucheriana*	8	12.5	0	0
		25	1	3
		50	4	4
		100	5	5
		200	7	6

## Conclusion

This study provides the scientific evidence that the extracts of *I. britannica* and *I. viscidula* are pharmacologically active which may be due to the existence of flavonoids, and they might be useful in the future treatment of epilepsy and sleep disorders. Since flumazenil antagonized the protective effects of the extracts from the seizure in animals, we can conclude that the extracts may interact with BZD receptors (23). Our study is still in progress and we are trying to isolate and identify the mechanisms of biologically active components of these important medicinal plants. 

## Compliance with Ethical Standards

All applicable international, national, and/or institutional guidelines for the care and use of animals were followed.

On behalf of all authors, the corresponding author states that there is no conflict of interest.

## Supplementary data

Below data provide number of animals, doses of extracts administered and number of protected animals 24 h following MES.
